# CRISPR Content Correlates with the Pathogenic Potential of *Escherichia coli*

**DOI:** 10.1371/journal.pone.0131935

**Published:** 2015-07-02

**Authors:** Enriqueta García-Gutiérrez, Cristóbal Almendros, Francisco J. M. Mojica, Noemí M. Guzmán, Jesús García-Martínez

**Affiliations:** Departamento de Fisiología, Genética y Microbiología. Universidad de Alicante, Campus de San Vicente, 03690 Alicante, Spain; SRI International, UNITED STATES

## Abstract

Guide RNA molecules (crRNA) produced from clustered regularly interspaced short palindromic repeat (CRISPR) arrays, altogether with effector proteins (Cas) encoded by cognate cas (CRISPR associated) genes, mount an interference mechanism (CRISPR-Cas) that limits acquisition of foreign DNA in *Bacteria* and *Archaea*. The specificity of this action is provided by the repeat intervening spacer carried in the crRNA, which upon hybridization with complementary sequences enables their degradation by a Cas endonuclease. Moreover, CRISPR arrays are dynamic landscapes that may gain new spacers from infecting elements or lose them for example during genome replication. Thus, the spacer content of a strain determines the diversity of sequences that can be targeted by the corresponding CRISPR-Cas system reflecting its functionality. Most *Escherichia coli* strains possess either type I-E or I-F CRISPR-Cas systems. To evaluate their impact on the pathogenicity of the species, we inferred the pathotype and pathogenic potential of 126 strains of this and other closely related species and analyzed their repeat content. Our results revealed a negative correlation between the number of I-E CRISPR units in this system and the presence of pathogenicity traits: the median number of repeats was 2.5-fold higher for commensal isolates (with 29.5 units, range 0–53) than for pathogenic ones (12.0, range 0–42). Moreover, the higher the number of virulence factors within a strain, the lower the repeat content. Additionally, pathogenic strains of distinct ecological niches (i.e., intestinal or extraintestinal) differ in repeat counts. Altogether, these findings support an evolutionary connection between CRISPR and pathogenicity in *E*. *coli*.

## Introduction

CRISPR-Cas systems are composed of at least one array of clustered regularly interspaced short palindromic repeats (CRISPR) and a set of *cas* (CRISPR-associated) genes [1,2]. Several CRISPR-Cas types (denoted I, II and III) and subtypes (identified with an additional letter) are distinguished according to the identity of the associated *cas* genes [3]. Although diverse tentative functions were initially postulated for particular systems [4–7], it has been demonstrated that they constitute an RNA-based interference mechanism that prokaryotes may utilize to avert infection by foreign genetic elements [8,9]. In brief, during encounters with invading DNA, short external sequences known as protospacers are integrated into a genomic CRISPR array through the acquisition process, becoming new repeat-intervening spacers [8,10–12]. This incorporation generally takes place at the end next to the leader [2,8,13–15], defined as an AT-rich sequence that usually, with the known exception of one type II system variant [16], governs transcription of the adjacent repeat-spacer array [14,17]. Afterwards, newly incorporated genetic elements with target regions matching spacer sequences will be degraded in the interference stage after annealing of the target with the complementary sequence in processed mono-spacer CRISPR RNA (crRNA) molecules [18,19]. These three main steps of CRISPR-Cas mechanism (spacer acquisition, crRNA processing and interference) require Cas proteins coded by the *cas* genes that are part of the system [19].

As a result of the diverse encounters that a cell lineage has experienced, the spacer content (number of spacers and their particular sequence) of a given CRISPR locus may vary greatly among closely related isolates. Moreover, the number of repeat-spacer units might be influenced by factors such as intrinsic acquisition activity, CRISPR-Cas expression levels or functionality of the Cas proteins in general [20–23]. Indeed, CRISPR-carrying strains that lack associated *cas* genes and/or leader show a reduced repeat number when compared to otherwise similar complete systems [15,21,23–25]. Furthermore, the acquisition efficiency in repeat arrays of a given CRISPR system varies in line with the leader expression level and repeat sequence conservation [26]. Thus, the complexity of a CRISPR array appears to mirror its overall activity.

CRISPR-Cas systems of I-E and I-F subtype may be found in *Escherichia coli*. However, some *E*. *coli* members lack the corresponding *cas* genes (*cas* I-E and I-F respectively) and only in very rare occasions are both simultaneously found [21,24]. Based on an early classification proposed by Kunin and coworkers [27], the CRISPR units of the I-E and I-F systems are assigned to clusters 2 and 4, respectively, of repeat types (here denoted CRISPR2 and CRISPR4). CRISPR2 are organized in *E*. *coli* in up to three arrays, accordingly named CRISPR2.1 (in CRISPR I locus, adjacent to the *cas* I-E genes), CRISPR2.2 and CRISPR2.3. The two latter arrays are located in the CRISPR II region, at a distance of 24 kb from CRISPR I. Occasionally a single array is found in CRISPR II, therefore called CRISPR2.2–3 [24]. Whereas CRISPR2.2 is constituted by 3 repeats and two invariable spacers, an analysis of 100 strains of the species disclosed up to a ten-fold difference (2–3 to 29–30) of repeat counts in CRISPR2.1 and CRISPR2.3 of systems with associated *cas* I-E genes [24]. Even though this diversity of CRISPR2 spacers is remarkable and the functionality of the I-E system has been demonstrated in a few *E*. *coli* strains [17,20,28,29], its role as a relevant genetic barrier in *E*. *coli* remains uncertain [24,28,30,31]. Referring to the I-F system, when the *cas* I-F genes are present, they are flanked by two CRISPR repeat arrays named CRISPR4.1 and CRISPR4.2 [24]. In contrast to I-E, these complete I-F systems have larger CRISPR arrays [24] and immunity to foreign elements has been detected under laboratory growth conditions without induction [20]. However, most *E*. *coli* strains lack *cas* I-F genes, then containing a single array (CRISPR4.1–2), with a reduced number of spacers.

A relation between CRISPR and pathogenicity has been illustrated by some remarkable observations in particular *E*. *coli* pathotypes and in other species. For example, a work demonstrated that CRISPR interference prevents acquisition of capsular virulence genes in *Streptococcus pneumoniae* [32]. Also, a link of CRISPR elements with serotypes and virulence potential of Shiga toxin-producing *E*. *coli* strains has been established [33]. However, the underlying cause of this association is unknown. In the context of the immunity role, we hypothesized that reduced CRISPR activity would pose fewer constraints to the entry of foreign genetic element and thus would favor lateral gene transfer (LGT). LGT events constitute one of the major driving forces in the evolution of prokaryotes [34–37]. Therefore, strains with limited immunity would be more prone to change their lifestyle [38], such as turning from commensal to pathogenic. Indeed, commensal *E*. *coli* (CEC) strains can become pathogens upon acquisition of virulence factors [39]. Moreover, infectivity of pathogenic strains could be enhanced after gaining more of these genes. In order to test whether the association between CRISPR and pathogenicity is a general trend in *E*. *coli*, and to shed light on the specific nature of such connection, the number of CRISPR repeat units in strains of *E*. *coli* and related species was compared with the presence of particular virulence genes involved in pathogenic processes [40,41]. Our results confirmed the CRISPR-pathogenicity association in *E*. *coli* and supported the defensive role of CRISPR as a driving force contributing to the emergence of pathogenic strains.

## Materials and Methods

### Strains and growth conditions

The microorganisms analyzed in this work comprise 126 strains (see S1 Table) harboring homologous CRISPR-Cas systems in equivalent locations [21]. These strains were chosen to cover a comprehensive range of commensal and pathogenic types, including intestinal (EnPEC) and extraintestinal (ExPEC) representatives. They consist of 124 *E*. *coli* and *Shigella* isolates, altogether referred here to as *E*. *coli* owing to the fact that both form a coherent phylogenetic group [21,42,43], and two strains of closely related species (*Escherichia fergusonii* ATCC35469 and *Escherichia albertii* TW07627). The 72 members of the ECOR collection [44], are included within the above mentioned panel of 124 *E*. *coli* isolates. Hereinafter, the remaining 54 strains will be collectively called non-ECOR. Full or almost completed genomes of these latter strains are available.

LB medium was typically used for growth of ECOR strains and incubations were carried out at 37°C for 12h with shaking. Sheep’s blood agar (bioMèrieux, Spain) was used to check hemolytic activity under the same temperature and time conditions.

### Pathotype ascription

ECOR strains that had not been previously identified as CEC or within a specific group of pathogenicity (i.e., pathotype), were subjected to hemolytic activity tests, a trait frequently linked to uropathogenic (UPEC) strains, and PCR screened, according to previous procedures [41,45], to assess the presence of genes usually associated with particular pathotypes (in brackets): *papG* (UPEC), *einv* (enteroinvasive *E*. *coli* or EIEC), *eaeA* (enteropathogenic *E*. *coli* or EPEC), *vt1* (enterohemorragic *E*. *coli* or EHEC), *lt1* (enterotoxigenic *E*. *coli* or ETEC) and *eagg* (enteroaggregative *E*. *coli* or EAEC). The amplification of any of the enteric markers (*einv*, *eaeA*, *vt1*, *lt1* or *eagg*) qualified for affiliation to EnPEC (as opposed to UPEC, here considered equivalent to ExPEC according to [39,46–48]), and the detection of just one of them was initially considered sufficient to categorize a strain within the respective pathotype. Furthermore, since *eaeA* can be additionally found in strains otherwise characterized as non-EPEC [49], those ECOR members yielding PCR amplifications of *eaeA* and the signature gene of another enteropathotype were ascribed to the latter. Apart from *eaeA*, if other EnPEC markers were observed within a strain, its specific pathotype was deemed as not conclusive and thus not considered for further analyses. Non-amplification of the signature gene of an EnPEC pathotype disqualified for ascription to it. In contrast, as uropathogenic strains frequently lack hemolytic activity and *papG*, their absence cannot be considered a sufficient criterion for exclusion from UPEC [39,45,47]. In consequence, when other uropathogenic determinants such as the *kps* or *sfa* operon (encoding capsule and S fimbriae respectively) had been reported [40], the strain was assumed to be UPEC.

Aside from the hemolytic activity usually linked to pathogenicity islands in UPEC, some EHEC strains can also carry a plasmid-encoded *hly* operon of similar sequence [39]. Thus, ECOR strains with the exclusive combination hemolysis-*vt1* were considered as EHEC. ECOR strains harboring other marker gene combinations associated with both EnPEC and ExPEC were assigned to the group with a higher representation of characteristic genes.

Among non-ECOR strains, only *Shigella* sp. D9 had not yet been categorized. In this case, computational searches of EnPEC and ExPEC determinants were performed to infer its affiliation.

Strains where pathogenic markers were not detected were considered as commensal.

### DNA extraction and polymerase chain reactions

DNA for sequencing and polymerase chain reactions (PCR) was extracted from 5 mL LB cultures grown as stated above. Cultures were centrifuged and pellets resuspended in 1 mL of ultrapure (milliQ) water for a total of three times. Cell suspensions were then lysed by heating at 98°C for 10 min and cell debris was removed by centrifugation. Finally, the supernatant solutions containing the DNA were stored at -20°C.

PCR amplifications performed to assess the pathogenic affiliation of ECOR strains were conducted with Taq polymerase (Roche) on a TC-3000 thermal cycler (Techne). Primers and conditions used are specified in S2 Table.

### Retrieval, processing and analysis of sequence data

The number of CRISPR units as well as the sequences of non-ECOR strains analyzed to assess the presence of genes involved in pathogenicity (i.e., *kps*, *hly*, *pap*, *sfa*, *einv*, *eaeA*, *vt1*, *lt1* and *eagg*) were obtained from previous works [21,24,41] or public databases (http://www.xbase.ac.uk/colibase/; http://www.ncbi.nlm.nih.gov/genomes/). CRISPR spacers were retrieved with CRISPRFinder [50] available at http://crispr.u-psud.fr/Server/, and similar sequences (over 75% identity) in non-CRISPR loci were searched with the CRISPRTarget tool [51] at http://bioanalysis.otago.ac.nz/CRISPRTarget/crispr_analysis.html.

For the phylogenetic analysis based on multilocus sequence typing (MLST), partial sequences from ECOR strains were downloaded from the Environmental Research Institute, University of Cork (http://MLST.ucc.ie; *dinB*, *icdA*, *pabB*, *polB*, *putP*, *trpA*, *trpB* and *uidA* genes) and from the Institut Pasteur (http://www.pasteur.fr/MLST; *adk*, *fumC*, *gyrB*, *icdA*, *mdh*, *purA*, and *recA* genes) web sites. In the case of non-ECOR strains, the same sets of sequences were retrieved from the abovementioned NCBI and XBASE sites. The concatenated sequence fragments from each strain were then aligned with CLUSTALW (http://align.genome.jp/) and a phylogenetic tree was constructed with the program MEGA version 6.06 (http://www.megasoftware.net/), using the UPGMA method with distances calculated by the Jukes-Cantor model on a pairwise-deletion comparison.

### Statistical analyses

Statistical analyses were performed with the SPSS software version 17.0 (SPSS 111 Inc., Chicago, IL). Kruskal-Wallis tests were used to infer differences in CRISPR counts. A *p*-value less than 0.05 was deemed as significant and validated the possible differences found for each of the corresponding groupings elaborated in this work of nonpathogenic or any of the pathogenic strains. Conversely, *p*-values higher than 0.05 were interpreted as proof of sufficient similarity among those groups compared. For robustness, these analyses were performed for groups with at least 3 strains.

To determine if significant correlations could be found, Pearson and Spearman coefficients (*r*) were calculated for the comparisons of different groups of strains with their respective CRISPR counts. In all cases, *p*-values lower than 0.05 were accepted for significance.

## Results

### Distribution of pathogenicity traits across *E*. *coli* and closely related species

As a first step for the comparison between CRISPR content and pathogenicity, strains under study were classified as either commensal or within a particular pathotype (see S1 Table). In the case of strains with a previously defined pathogenic profile, the ascription reported was adopted. Otherwise, the pathotype of *Shigella* sp. D9 and those ECOR strains not previously characterized was inferred following the criteria described in Materials and Methods. The robustness of these criteria was demonstrated by the high degree of coincidence between the pathotype described for categorized strains and the one predicted after the detection of the selected pathogenicity markers in the genomes of such strains (S1 Table). Seeming exceptions in EnPEC genomes were the *E*. *coli* strains P12b and 101.1, previously assigned to EPEC and EAEC respectively, where we did not find the corresponding markers (*eaeA* and *eagg*). Nevertheless, these results were in agreement with reports for other strains [49,52–55], indicating that *eaeA* and *eagg* might not be considered as signatures invariably linked to the respective pathogenic group. In the case of the UPEC/ExPEC strains, our marker-based ascriptions were also highly coincident with pathogenicity documented. The most striking difference involved strain EC23, which showed hemolytic activity (encoded by the *hly* operon) in our tests and *papG* was amplified, even though these UPEC genes had not been detected in a previous Southern analysis [40]. This inconsistency might be due to low sequence conservation in this strain of the probes used in the Southern blot analyses. Another somehow unexpected result was the finding of some UPEC traits in several strains that had been deemed to be CEC or EnPEC (S1 Table), which could be attributed to the great genome plasticity found in *E*. *coli* and the fact that genes, while present, may not necessarily be expressed [56,57]. This prompted us to ascribe pathogenicity solely based on the nature and number of the ExPEC or EnPEC virulence traits.

### Comparison of repeat content with pathogenicity

**Fig 1:**
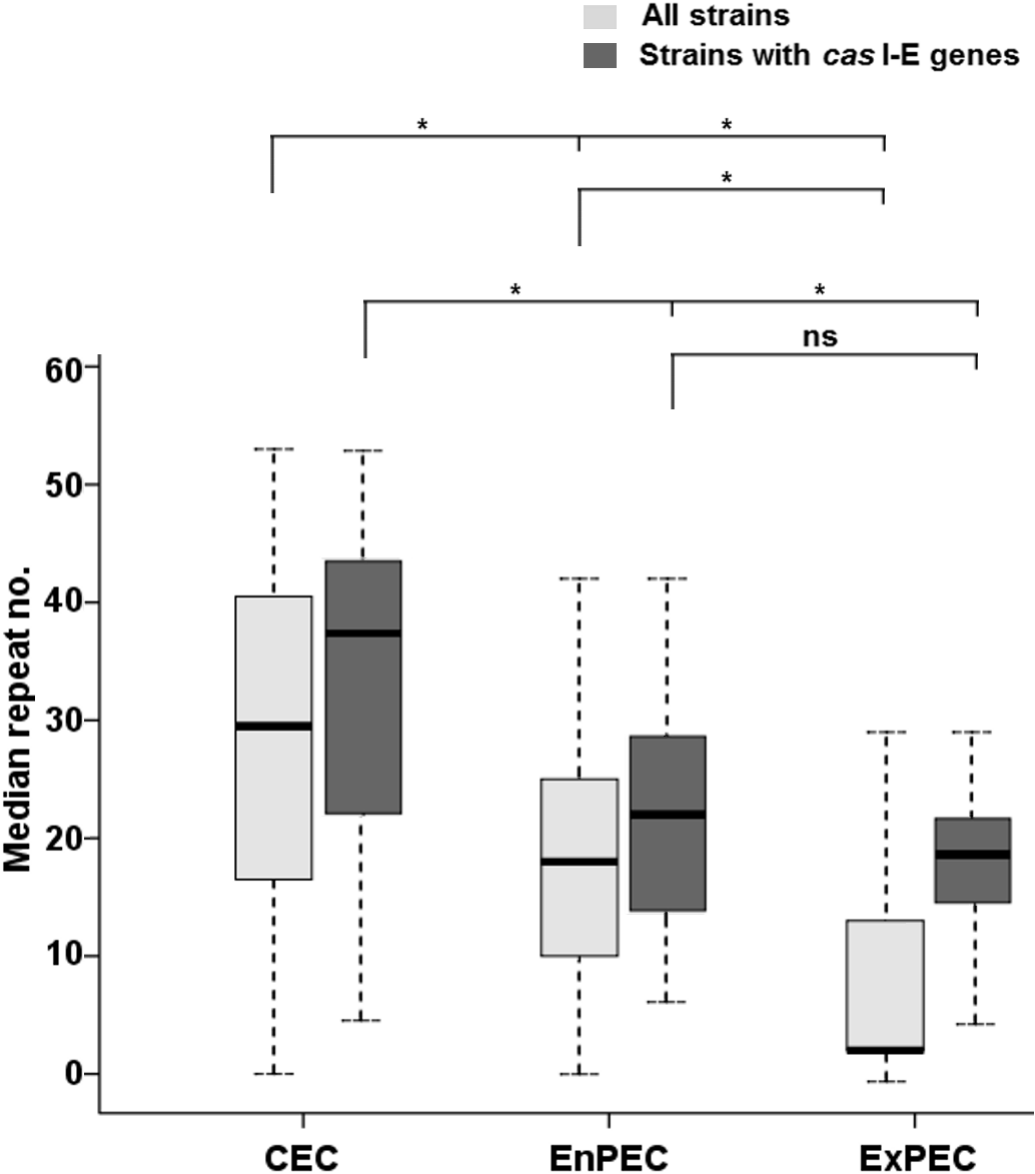
Comparison of CRISPR counts and pathogenic categories. Median numbers of CRISPR2 units in commensal (CEC), enteric (EnPEC) or extraintestinal (ExPEC) pathogens of the *E*. *coli* and related strains analyzed in this study, are indicated by a horizontal line. Light grey boxes represent the interquartile range values for the whole set of 126 strains (with 28, 50 and 43 isolates for each group, respectively). Dark grey boxes comprise the interquartile range values for the reduced subset of 71 strains with intact *cas* I-E genes (22, 35 and 11 isolates). Vertical lines for each box denote the corresponding CRISPR2 count range. Significant differences of median values (Kruskal-Wallis *p*-values lower than 0.05) for the comparisons within each of these two sets of strains are indicated by an asterisk (ns, not significant).

**Fig 2:**
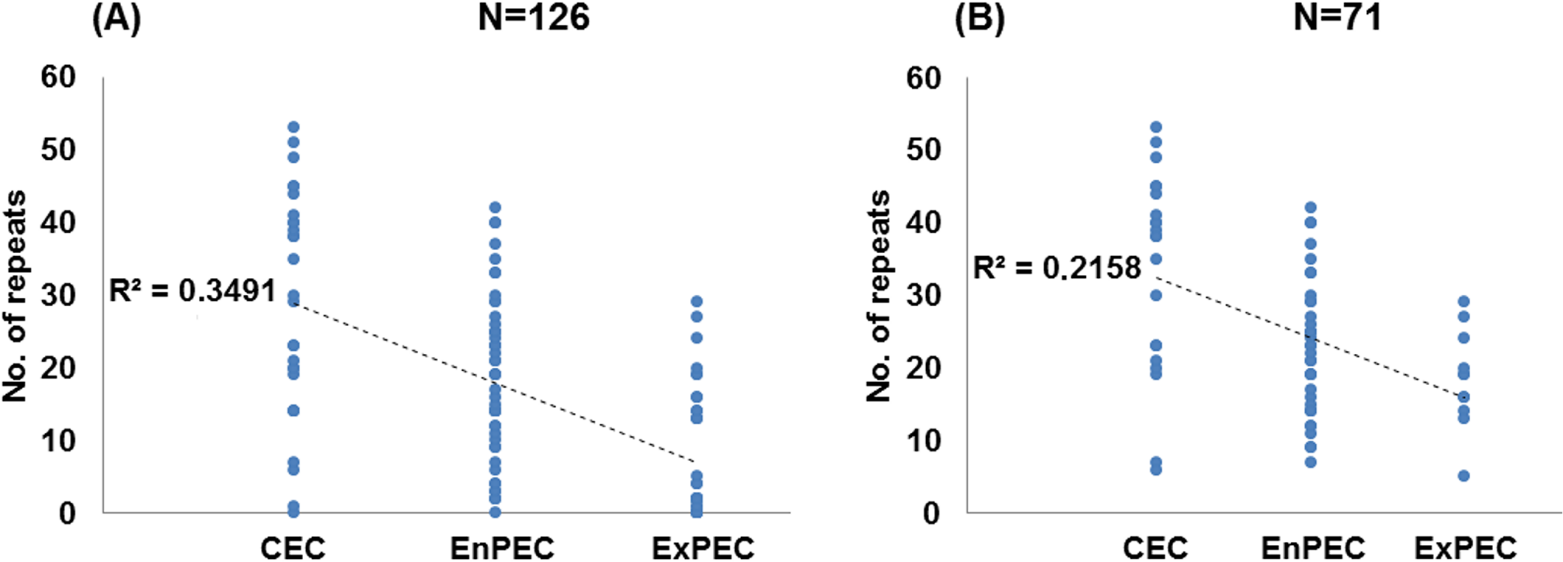
Correlation of CRISPR counts and pathogenic categories. Graphical representation of the number of CRISPR repeats in strains categorized as commensal (CEC) or as pathogens of enteric (EnPEC) or extraintestinal (ExPEC) origins for the whole set of N = 126 strains (A) or the 71 strains with the intact set of *cas* I-E genes (B). Dotted lines represent the least-square linear regressions, and their corresponding R^2^ values are indicated.

Once strains were catalogued as commensal or with a specific pathotype, this profile was compared with the number of CRISPR2 repeats (see S1 Table) and statistical analyses were conducted. A strong negative correlation was found between the CRISPR2 repeat count and the possession of pathogenic traits (Pearson’s *r* = -0.465, with *p* = 0.01 for comparison A of all strains in S1 Table). Generally, the median number of repeats for CEC strains was higher than for pathogenic strains (29.5 vs. 12.0 with *p* = 0.000; see comparison A for all strains in Table 1). Moreover, differences in the count of CRISPR2 units were also observed between ExPEC and EnPEC. In accordance with previous results [58], ExPEC pathogens usually carried fewer repeats than CEC. Furthermore, this number was lower than for EnPEC strains (2 in ExPEC compared to 18 in EnPEC; see Fig 1, S1 Table and comparison B in Table 1, N = 126), with a Pearson’s correlation coefficient of *r* = -0.591 for a significance value of *p* = 0.01 (Fig 2A). In contrast, differences in repeat numbers for the diverse EnPEC pathotypes were not significant (*p*>0.07, comparison C in Table 1, N = 126). Furthermore, no statistically significant distinction (*p* = 0.887) could be made between ECOR strains carrying enteric markers and non-ECOR EnPEC members (comparison D in Table 1, N = 126). This equivalence between both sets of strains confirmed the overall validity of our PCR analyses. However, it should be noted that range values (minimum and maximum no. of CRISPR units) within each group considered in Table 1 were larger than those found in similar studies [33,58]. This hints to a higher strain diversity within the groups considered in this work (see discussion).

**Table 1:** Groups of strains studied for which statistical comparisons of repeat content and pathogenicity were performed.

		All strains (N = 126)			Strains with intact I-E genes (N = 71)		
Comparison	Group of strains compared	No. of strains	Median repeat no.	Repeat no. range	No. of strains	Median repeat no.	Repeat no. range
A	1. Commensal	28	29.5	0–53	22	38.0	6–53
	2. Pathogenic	98	12	0–42	49	21.0	5–42
B	1. Commensal	28	29.5	0–53	22	38.0	6–53
	2. EnPEC	50	18	0–42	35	23.0	11–42
	3. ExPEC	43	2	0–29	11	19.0	5–29
C	1. EIEC	9	10	3–42	3	24.0	15–42
	2. EPEC	10	14	0–33	6	17.5	9–33
	3. ETEC	5	23	12–40	4	26.0	22–40
	4. EHEC	17	21	6–35	16	25.0	7–35
D	1. EnPEC (ECOR)	41	13	0–42	23	25.0	2–42
	2. EnPEC (non-ECOR)	31	14	0–40	18	19.0	2–40
E	1. n = 0 UPEC genes^a^	63	21	0–53	49	26	6–53
	2. n = 1 UPEC genes^a^	23	13	0–29	12	20.5	7–29
	3. n = 2 UPEC genes^a^	22	2	0–31	9	19	14–31
	4. n = 3 UPEC genes^a^	7	2	1–5	1	5	-
	5. n = 4 UPEC genes^a^	11	2	0–2	0	-	-

^a^Strains with the same total number of UPEC factors considered in the study.

The inclusion in this study of strains lacking *cas* I-E genes (hence with a similarly reduced repeat number) might generate distorted results due to a possible clonal effect. However, when comparisons were performed for the subset of 71 strains carrying a complete set of *cas* I-E, the results were highly coincident with those obtained for all strains (Table 1). The only exception corresponded to the lack of discrimination (*p* = 0.172) between EnPEC and ExPEC (Fig 1, S1 Table and comparison B in Table 1, N = 71). However, strong negative correlation values were still found between repeat numbers and pathotype (Pearson’s *r* = -0.465, with *p* = 0.01, see Fig 2B). These results with the purged set of 71 strains suggest that *cas* I-E functionality, rather than a phylogenetic (i.e. clonal) constraint, would be the main cause of the relationship found between CRISPR and pathogenicity. To provide further support to this conclusion, the distribution within phylogroups A and B1 of pathogenic and commensal strains with a complete set of *cas* I-E genes was analyzed [21]. These two phylogenetically related MLST groups were selected for the analysis since they include the majority of *cas* I-E harboring strains (N = 52). The results obtained showed that CEC and pathogenic strains were present across all the major phylogenetic subgroups within A and B1 (S1 Fig). In spite of this scattered distribution, a negative correlation (see S2 Fig) could still be observed when comparing CEC, EnPEC and ExPEC with their CRISPR repeat counts, with a Pearson coefficient of *r* = -0.476 for a significance of *p* = 0.01. This observation in strains sharing the same phylogenetic constraints further hints that CRISPR-Cas systems may influence, at least partially, on pathogenicity.

In the case of the I-F system, the associated *cas* genes were only detected in 14 strains of those under study, the majority being pathogenic (S1 Table). This suggested a much reduced impact on pathogenicity of I-F compared to I-E.

### Higher numbers of uropathogenicity genes relate to lower repeat counts

**Fig 3:**
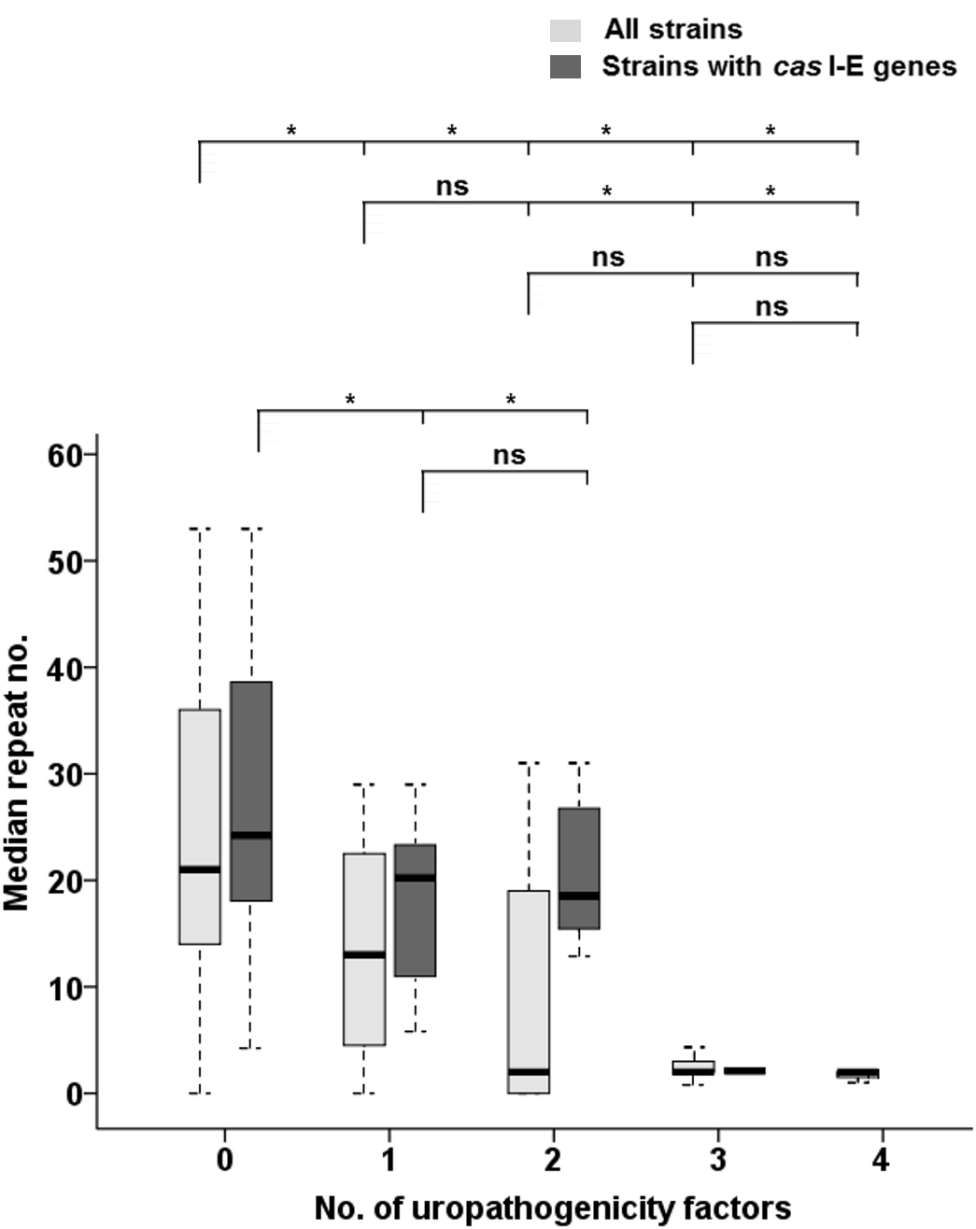
Comparison of the CRISPR counts and the number of UPEC genes. Median numbers of CRISPR2 units in the strains under study, referred to the number of selected uropathogenicity genes within those strains. For each UPEC number category (x-axis), light grey boxes represent the interquartile range for the median value (horizontal line) of all strains (N = 126, with 63, 23, 22, 7 and 11 isolates for each category, respectively), while dark grey boxes indicate that value for strains with complete *cas* I-E genes (N = 71 and 49, 12, 9, 1 and 0 isolates, respectively). Vertical lines indicate the CRISPR2 count ranges. Significant differences of median values (Kruskal-Wallis *p*-values lower than 0.05) for the comparisons within each set of strains are indicated by an asterisk (ns, not significant). The categories compared are indicated in brackets, while categories with an insufficient number of isolates are not considered for comparison (see Materials and Methods).

**Fig 4:**
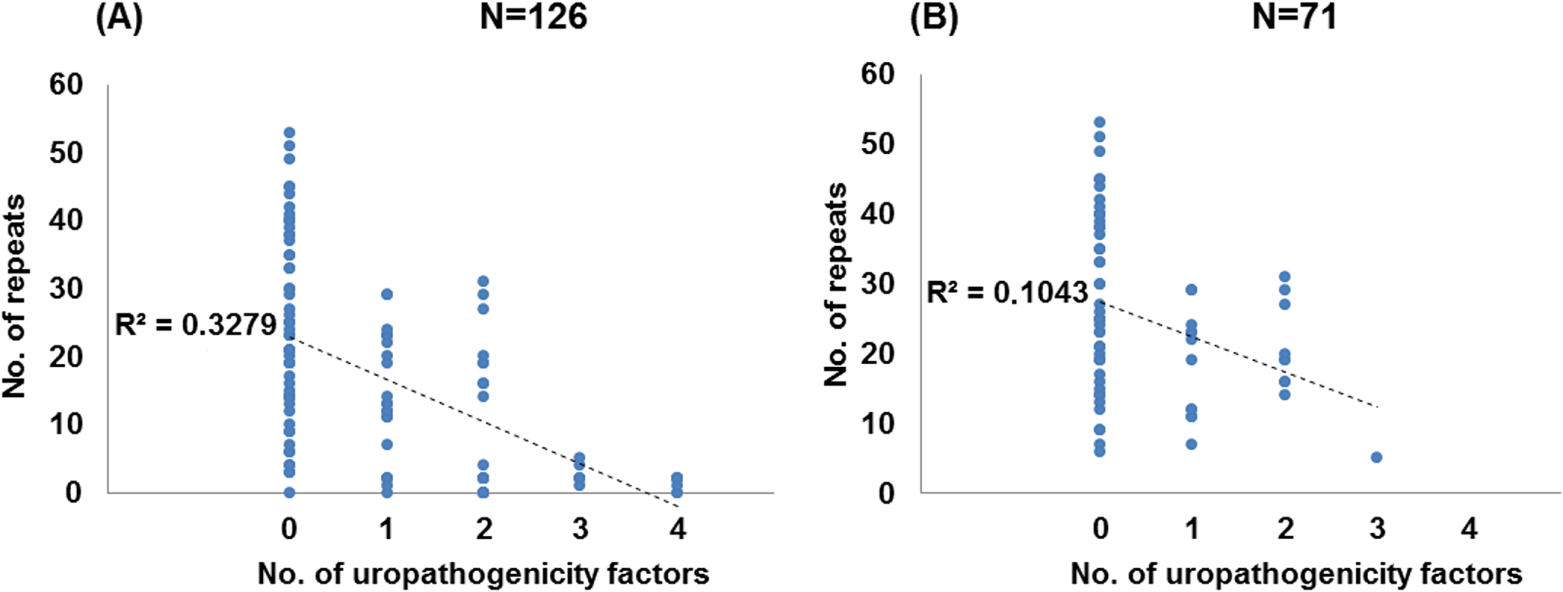
Correlation of CRISPR counts and the number of UPEC genes. Graphical representation of the number of CRISPR repeats for strains harboring 0, 1, 2, 3 or 4 UPEC factors for the whole set of N = 126 strains (A) or the 71 strains with the intact set of *cas* I-E genes (B). Dotted lines represent the least-square linear regressions, and their corresponding R^2^ values are indicated.

In contrast to EnPEC pathotypes where just one pathogenicity factor was considered in this study, a total of four markers were probed for UPEC. This allowed us to perform an analysis in this latter case to assess a correlation between the repeat count and the number of such pathogenic traits within each strain. This analysis showed that, regardless of their classification as pathogen or commensal, strains with the lowest number of repeats tended to bear more of such factors (Fig 3, S1 Table and comparison E in Table 1, N = 126), showing a strong negative correlation (Spearman’s *r* = -0.622, *p* = 0.01, see Fig 4A). Furthermore, strains in possession of 1 uropathogenic determinant had six times more CRISPR units than those carrying 2 or more (Fig 3, S1 Table and comparison E in Table 1, N = 126), ranging from 13 repeats (1 factor) to 2 (2–4 factors). This suggested a relationship between CRISPR activity and the capability to incorporate such pathogenic factors. Thus, it could be inferred that a greater virulence potential (in terms of a higher number of factors) is associated with lower repeat counts. However, while Kruskal-Wallis tests differentiated (in terms of CRISPR count) between strains with 1 or no UPEC factors from the rest, they did not discriminate between strains with 2, 3 or 4 UPEC factors (*p*>0.05 in all cases, see Fig 3). This lack of differentiation might suggest a certain degree of specialization at least in uropathogenicity, where a critical number of virulence determinants should be required to elicit pathogenicity. This conclusion is further supported when considering that, of the 16 strains with a previously defined pathotype that were in possession of just 1 UPEC factor (see S1 Table), only in 2 was the reported pathotype UPEC/ExPEC, whereas in the rest was either CEC (4 strains) or EnPEC (10 strains). In contrast, of the 20 previously ascribed strains carrying 2 to 4 UPEC factors, 19 had been deemed as uropathogens [44,59–63].

When strains without *cas* I-E genes were purged, an almost 4-fold difference in repeat counts between strains with 1–2 versus 3 factors (19 vs 5, S1 Table and comparison E in Table 1, N = 71) was observed, with strong negative correlation values (Spearman’s *r* = -0.320, with *p* = 0.01, see Fig 4B). Nevertheless, the fact that just one isolate contained 3 factors did not allow us to assess significance for all the groups compared, albeit *p* = 0.007 was obtained to differentiate between strains carrying no UPEC determinants and those with at least one of them (Fig 3). These results for the 71 strains, coupled with those from the same subset regarding CEC, EnPEC and ExPEC groupings, strongly suggest that loss of CRISPR activity allowed ExPEC specialization, and that this loss was more often accomplished by the removal of the *cas* I-E genes.

### Correlation between CRISPR-Cas I-E repeat numbers and pathogenicity in other *Escherichia* species

The *E*. *fergusonii* ATCC35469 and *E*. *albertii* TW07 strains included in this study showed the general pattern of correlation between pathogenicity and CRISPR counts observed in *E*. *coli* (S1 Table). Thus, the commensal *E*. *fergusonii* strain ATCC35469 [64] has a number of repeat units (n = 38) within the range of the median values found for CEC (n = 29.5 or n = 38, depending on the set comprising all strains or the one purged of *cas*-less strains, respectively), and the CRISPR unit count in the enteropathogenic *E*. *albertii* TW07627 [65] is on par with the median values encountered in the EnPEC isolates. Taken together, these results further support a link between the I-E CRISPR-Cas system and the pathogenicity of *E*. *coli*-related microorganisms.

## Discussion

### Impact of the I-E CRISPR-Cas system on the pathogenicity of *Escherichia*

A negative correlation has been established in this work between the repeat content in the I-E system and the pathogenicity of *E*. *coli* and related strains. However, several explanations could account for this relationship. In principle, it could be interpreted as the consequence of the immunity role of CRISPR: those systems with higher numbers of spacers, as a result of a higher mean activity [26], will act as more efficient barriers against invaders, such as those carrying virulence factors that promote pathogenicity [39,46,64,66]. Although the immune function has been proven in other species, the apparently low dynamics of the CRISPR arrays of *E*. *coli* suggests that they do not act as would be expected for an efficient barrier [30]. Nevertheless, the low turnover of spacers should be seen as a consequence of the stringent regulation that governs expression of CRISPR-Cas I-E [17,67–70], being silenced under normal growth conditions [17,67]. Moreover, laboratory strains are able to elicit CRISPR-mediated interference against plasmids and phages [69,71] and the widespread presence in *E*. *coli* strains of spacers with identities to viral and plasmid sequences [24] strongly supports the defense role of CRISPR-Cas. Indeed, a search for spacer homologs revealed that 98 out of the 114 strains studied harboring spacers have at least one that matches sequences in transmissible elements (S1 Table).

A previous work on *E*. *coli* reported no meaningful association between the presence in the cell of *cas* I-E genes and that of plasmids [31], arguing against a role of the I-E system as a barrier to the import of a genetic element. However, I-E spacers target mainly phages, with a relatively low proportion of plasmids [20], with a ca. five to one ratio for these elements, respectively (see S1 Table). These results suggest that I-E would preferentially limit viruses and, in the context of pathogenicity, CRISPR would be mainly hindering acquisition of virulence factors carried by these infectious elements. By contrast, the phage-plasmid ratio of spacer homologs in those strains carrying the less prevalent I-F is 24 to 43, albeit 15 of the plasmid homologs are found within a single CEC isolate (strain ED1a, see S1 Table). Remarkably, ED1a and *Shigella* sp. D9 are the only CEC strains carrying I-F whereas the rest are pathogenic. In this sense, it should be noted that, whereas some of the EnPEC markers considered in this work (namely *einv* and *eagg*) may be carried by plasmids, they are also present as part of chromosomal pathogenicity islands which, due to their size, are usually located within prophages or in association with transposons [39]. Thus, the potential association of I-F on pathogenicity, despite being more active than I-E [20] seems, due to its affinity to genetic elements and low prevalence, more negligible than I-E.

An alternative explanation for the CRISPR-pathogenicity association is that the I-E system may be related to regulation of expression of virulence genes, as has been seen in other microorganisms where Cas proteins enable or increase pathogenicity [72,73]. However, if a regulatory involvement would apply to the *E*. *coli* systems, such role should be as a repressor rather than inducer (less active system in more pathogenic strains). Moreover, repeat counts should not be directly related to this activity [72]. Thus, the variations in the number of repeat-spacer units must reflect foreign attacks (immunization), and consequent targeting activity rather than regulation of virulence factors.

These findings suggest that CRISPR activity may have hindered the emergence of pathogenic lifestyles in *E*. *coli* [73]. Alternatively, our results could be interpreted the other way around: that the pathogenic behavior promoted a reduced activity of CRISPR-Cas elements. However, the ancestral presence in *Escherichia* of the CRISPR systems, altogether with the absence of *cas* genes in pathogenic groups, notably of I-E subtype in the B2 group of MLEE strains [21], disputes the latter possibility. Regarding *E*. *coli* phylogeny, the subset of strains with functional I-E systems, which mainly belong to closely related MLEE groups A and B1 [21], follows the same correlation of repeat counts and pathogenicity (as mentioned above). This fact should be considered as another indication of the role of CRISPR activity on pathogenicity, as opposed to the repeat distribution being merely the result of a phylogenetic constraint.

### Relationship between habitat and CRISPR-Cas activity

In the context of CRISPR acting as an immune system, differences in its activity among strains would be expected, for instance due to genetic diversity or the varied inducing factors they encounter in their respective habitat. These factors include the frequency they face invaders, the diversity of such invaders or the occurrence of mutations in the target that will prompt efficient acquisition [26,71,74,75]. Certainly, a link between the habitat to which the strains adapt and CRISPR activity is supported by the differences we found in the repeat content between intestinal and extraintestinal strains. However, CEC strains carry a significantly higher number of repeats than EnPEC, even though the members of both groups share habitat, being confined almost exclusively within the gut. This difference in repeat counts could be explained by a different frequency of successful events of lateral gene transfer (LGT) in commensal and pathogenic strains. Indeed, the gut is a bacteriophage-rich environment [76,77], where strong selective pressure must exist favoring the occurrence of efficient mechanisms preventing phage infection. Nevertheless, taking into account that phages are also an important source of virulence factors, it is expected that EnPEC strains will have more permissive (i.e., less active) defense systems against these infective agents than CEC.

In the case of ExPEC strains, which also colonize secondary habitats where viral predators are scarcely present [78,79], less selective pressure together with the above stated advantage for a pathogen to allow LGT, would justify a further reduction in CRISPR activity.

### CRISPR count diversity reveals a notable heterogeneity of pathogenic populations of *E*. *coli*

The large interquartile ranges of many CRISPR counts that were found within CEC and each of the pathotypes (both in ECOR and non-ECOR strains) suggested the existence of very diverse populations within each group. Several reasons could account for such dispersion. For instance, the contribution of barriers alternative to CRISPR-Cas, which may compensate a reduced CRISPR activity (i.e., low repeat counts) in some commensal strains. Similarly, pathogenic strains may possess exceptionally active CRISPRs that would counterbalance the lack of alternative barriers. Nevertheless, an inaccurate ascription of some strains within each group (e.g. some pathogenic strains having been deemed to be commensal or vice versa) cannot be dismissed. Indeed, this categorization is error-prone as pathogenicity is a complex process. Among others, factors such as medical procedures performed on patients, their general health status, the molecular affinity of microbial pathogenic gene products for a specific host, and hence different levels of virulence could alter the outcome to either pathogenic or commensal [80–82]. Otherwise, in the case of strains where an established pathogenicity profile was not available, we inferred it by the presence of traits characteristic of a specific pathotype. Nevertheless, the presence of a particular trait does not determine pathogenicity, since it might not be functional [56]. Moreover, as observed here in the case of UPEC strains, true pathogenicity might require a certain critical number of virulence traits. This biased marker-based ascription might certainly account for at least some of the apparent intra-pathogroup diversity encountered.

## Conclusions

A correlation has been established linking a reduced repeat content in the I-E system of *Escherichia coli* and related strains with a higher probability for a specific strain to exert pathogenicity (i.e. the potential ability of a microorganism to cause disease). Moreover, significant differences in the CRISPR count also correlate with the environment in which this pathogenicity is performed, despite all strains normally reside in the gut. However, the great variability in the number of CRISPR units for strains within a pathogenic group would make its potential application for predictive studies of pathogenicity best suited as supplementary to other techniques. The increase in genomic data and a more accurate characterization of the strains (*E*. *coli* and other species) in terms of their pathogenic profile and their particular CRISPR-Cas activity will provide new clues to better understand this correlation. Nevertheless, the influence of CRISPR-Cas as a barrier regulating the influx of LGT, and the subsequent impact on the diversity of *E*. *coli* and related species, should be a factor to be considered to better understand gene exchange phenomena from an evolutionary standpoint.

## Supporting Information

S1 Fig Phylogenetic distribution of commensal and pathogenic strains. Tree showing the MLST relationships corresponding to the strains analyzed in this study belonging to phylogroups A and B1 (see Almendros *et al*., 2014). Only isolates that carry a complete set of *cas* I-E genes are considered. CEC, EnPEC and ExPEC strains are indicated in green, blue and red, respectively. EC58, in black, is a potentially pathogenic strain not assigned to EnPEC or ExPEC (see S1 Table). Strain *Escherichia fergusonii* ATCC35469 was used as outgroup (branch length, truncated, not to scale).
(TIF)

S2 Fig Correlation of CRISPR counts and pathogenic categories of strains in MLST groups A and B1. Graphical representation of the number of CRISPR repeats in strains categorized as commensal (CEC) or as pathogens of enteric (EnPEC) or extraintestinal (ExPEC) origin. The strains analyzed (N = 52) belong to phylogroups A and B1 and carry a complete set of *cas* I-E genes. A dotted line represents the least-square linear regression. The R^2^ value is indicated.
(TIF)

S1 Table Strain data of CRISPR counts, spacer homologs, presence of *cas* I-E genes, pathogenic traits and pathogenicity categories. (XLS)

S2 Table Primers and conditions used for amplification of pathogenicity markers. (DOC)
