# Correction: CRISPR Content Correlates with the Pathogenic Potential of *Escherichia coli*

**DOI:** 10.1371/journal.pone.0134138

**Published:** 2015-07-28

**Authors:** The PLOS ONE Staff

The following information is missing from the Funding section: This study was supported by grant BIO2014-53029 from the Ministerio de Economía y Competitividad (http://www.mineco.gob.es/portal/site/mineco/idi). The publisher apologizes for the error.
