# Supplementary figures and images for: CRISPR Content Correlates with the Pathogenic Potential of Escherichia coli
Source: PLoS One. 2015 Jul 2;10(7):e0131935. doi: 10.1371/journal.pone.0131935 (PMC4489801; doi:10.1371/journal.pone.0131935)

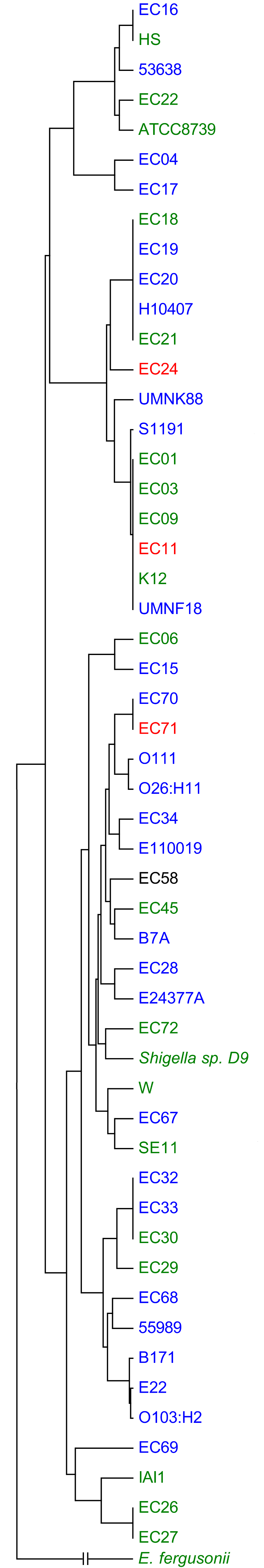

Supplement: S1 Fig — Tree showing the MLST relationships corresponding to the strains analyzed in this study belonging to phylogroups A and B1 (see Almendros et al., 2014). Only isolates that carry a complete set of cas I-E genes are considered. CEC, EnPEC and ExPEC strains are indicated in green, blue and red, respectively. EC58, in black, is a potentially pathogenic strain not assigned to EnPEC or ExPEC (see S1 Table). Strain Escherichia fergusonii ATCC35469 was used as outgroup (branch length, truncated, not to scale). (TIF) [file pone.0131935.s001.tif]

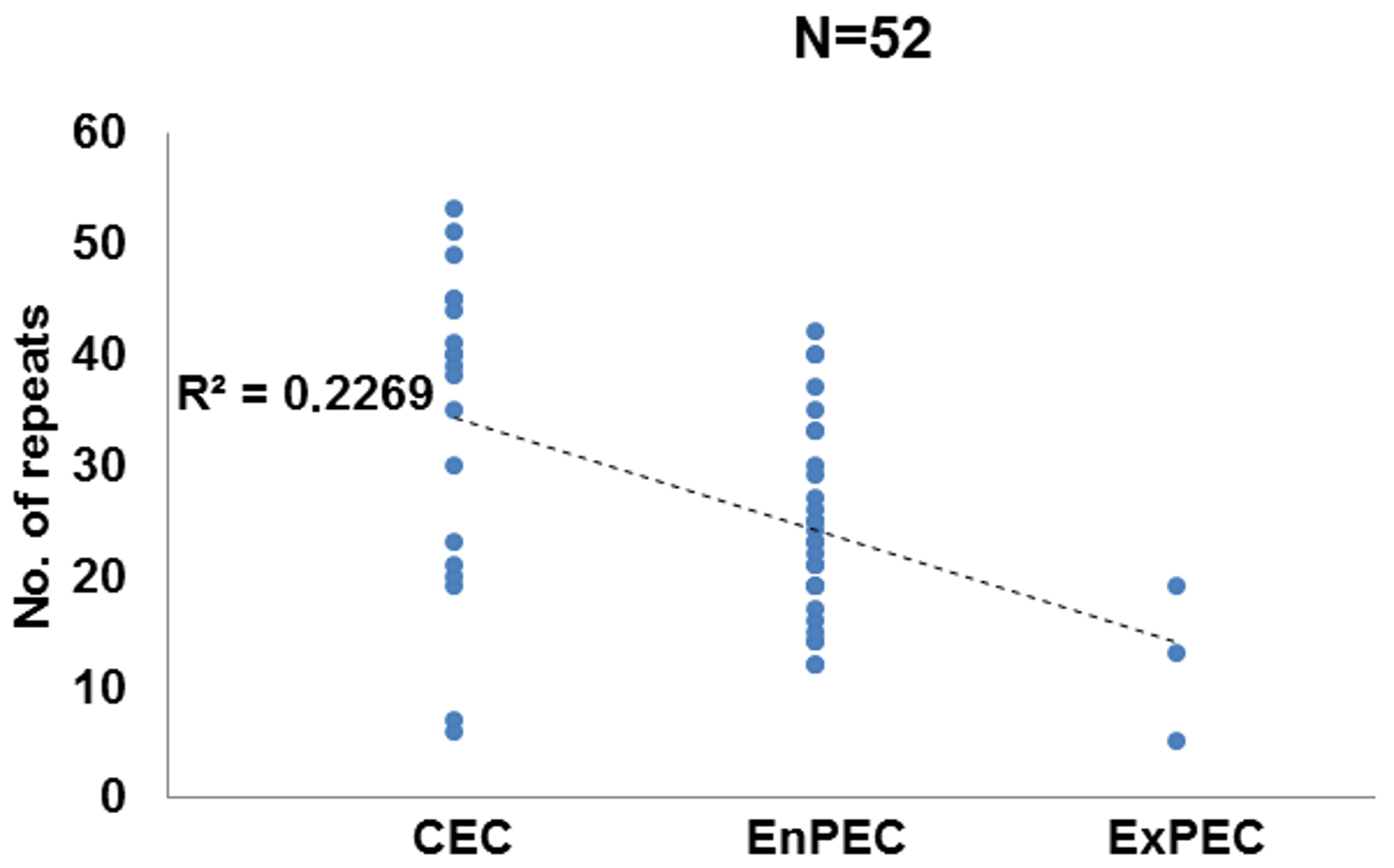

Supplement: S2 Fig — Graphical representation of the number of CRISPR repeats in strains categorized as commensal (CEC) or as pathogens of enteric (EnPEC) or extraintestinal (ExPEC) origin. The strains analyzed (N = 52) belong to phylogroups A and B1 and carry a complete set of cas I-E genes. A dotted line represents the least-square linear regression. The R2 value is indicated. (TIF) [file pone.0131935.s002.tif]
