# Supplementary material for: CRISPR Content Correlates with the Pathogenic Potential of Escherichia coli
Source: PLoS One. 2015 Jul 2;10(7):e0131935. doi: 10.1371/journal.pone.0131935 (PMC4489801; doi:10.1371/journal.pone.0131935)
Supplement: S2 Table — (DOC) [file pone.0131935.s004.doc]

**S2 Table. Primers and conditions used for amplification of pathogenicity markers.**

| **Primer** | **5’-3’ sequence (target)** | **PCR conditions** |
| --- | --- | --- |
| papG-F | CTGTAATTACGGAAGTGATTTCTG (types II and III adhesin G genes in uropathogenic strains) | Denaturing: 95^o^C, 10s.  Annealing: 60^o^C, 15s.  Extension: 72^o^C, 40s.  No. cycles: 35. |
| papG-R | CACTCTCCGGCTCCGGATAAACCAT (types II and III adhesin G genes in uropathogenic strains) | Idem |
| einv-F | TGGAAAAACTCAGTGCCTCTGCGG (fragment of *einv* in enteroinvasive strains) | Denaturing: 95^o^C, 30s.  Annealing: 72^o^C, 30s.  Extension: 72^o^C, 30s.  No. cycles: 5.  Denaturing: 95^o^C, 30s.  Annealing: 60^o^C, 30s.  Extension: 72^o^C, 30s.  No. cycles: 30. |
| einv-R | TTCTGATGCCTGATGGACCAGGAG (fragment of *einv* in enteroinvasive strains) | Idem |
| eaeA-F | TGAGCGGCTGGCATGAGTCATAC (fragment of *eaeA* in enteropathogenic strains) | Idem |
| eaeA-R | TCGATCCCCATCGTCACCAGAGG (fragment of *eaeA* in enteropathogenic strains) | Idem |
| vt1-F | ACGTTACAGCGTGTTGCTGGGATC (fragment of *vt1* in enterohemorragic strains) | Idem |
| vt1-R | TTGCCACAGACTGCGTCAGTTAGG (fragment of *vt1* in enterohemorragic strains) | Idem |
| lt1-F | TGGATTCATCATGCACCACAAGG (fragment of *lt1* in enterotoxigenic strains) | Idem |
| lt1-R | CCATTTCTCTTTTGCCTGCCATC (fragment of *lt1* in enterotoxigenic strains) | Idem |
| eagg-F | GACTCTGGCGAAAGACTGTATC (fragment of *eagg* in enteroaggregative strains) | Idem |
| eagg-R | ATGGCTGTCTGTAATAGATGAGAAC (fragment of *eagg* in enteroaggregative strains) | Idem |
